# Modulation of Vagal Sensory Neurons *via* High Mobility Group Box-1 and Receptor for Advanced Glycation End Products: Implications for Respiratory Viral Infections

**DOI:** 10.3389/fphys.2021.744812

**Published:** 2021-09-21

**Authors:** Stuart B. Mazzone, Seung-Kwon Yang, Jennifer A. Keller, Juste Simanauskaite, Jaisy Arikkatt, Matthew J. Fogarty, Aung Aung Kywe Moe, Chen Chen, Matthew W. Trewella, Luyi Tian, Matthew E. Ritchie, Brendan Y. Chua, Simon Phipps, Kirsty R. Short, Alice E. McGovern

**Affiliations:** ^1^Department of Anatomy and Physiology, The University of Melbourne, Parkville, VIC, Australia; ^2^School of Biomedical Sciences, The University of Queensland, Brisbane, QLD, Australia; ^3^Molecular Medicine Division, The Walter and Eliza Hall Institute of Medical Research, Parkville, VIC, Australia; ^4^The Peter Doherty Institute for Infection and Immunity, Department of Microbiology and Immunology, University of Melbourne, Melbourne, VIC, Australia; ^5^QIMR Berghofer Medical Research Institute, Herston, QLD, Australia; ^6^School of Chemistry and Molecular Biosciences, The University of Queensland, Brisbane, QLD, Australia

**Keywords:** vagal ganglia, visceral sensory, hypersensitivity, respiratory virus, hyperinnervation

## Abstract

Vagal sensory neurons contribute to the symptoms and pathogenesis of inflammatory pulmonary diseases through processes that involve changes to their morphological and functional characteristics. The alarmin high mobility group box-1 (HMGB1) is an early mediator of pulmonary inflammation and can have actions on neurons in a range of inflammatory settings. We hypothesized that HMGB1 can regulate the growth and function of vagal sensory neurons and we set out to investigate this and the mechanisms involved. Culturing primary vagal sensory neurons from wildtype mice in the presence of HMGB1 significantly increased neurite outgrowth, while acute application of HMGB1 to isolated neurons under patch clamp electrophysiological investigation produced inward currents and enhanced action potential firing. Transcriptional analyses revealed the expression of the cognate HMGB1 receptors, Receptor for Advanced Glycation End products (RAGE) and Toll-like Receptor 4 (TLR4), in subsets of vagal sensory neurons. HMGB1-evoked growth and electrophysiological responses were significantly reduced in primary vagal sensory neurons harvested from RAGE deficient mice and completely absent in neurons from RAGE/TLR4 double deficient mice. Immunohistochemical analysis of vagal sensory neurons collected from mice after intranasal infection with murine pneumovirus or influenza A virus (IAV), or after intratracheal administration with the viral mimetic PolyI:C, revealed a significant increase in nuclear-to-cytoplasm translocation of HMGB1 compared to mock-inoculated mice. Neurons cultured from virus infected wildtype mice displayed a significant increase in neurite outgrowth, which was not observed for neurons from virus infected RAGE or RAGE/TLR4 deficient mice. These data suggest that HMGB1 can enhance vagal sensory neuron growth and excitability, acting primarily *via* sensory neuron RAGE. Activation of the HMGB1-RAGE axis in vagal sensory neurons could be an important mechanism leading to vagal hyperinnervation and hypersensitivity in chronic pulmonary disease.

## Introduction

The airways and lungs are densely innervated by sensory neurons, the majority of which are derived from the nodose and jugular vagal sensory ganglia (Mazzone and Undem, 2016). These bronchopulmonary sensory nerves serve to monitor the local airway and pulmonary environment and when activated, evoke reflexes and behaviors important for regulating pulmonary defense, airway patency, respiration, and pulmonary inflammatory responses (Mazzone and Undem, 2016; McGovern et al., 2018). In many pulmonary diseases, bronchopulmonary sensory nerve fibers undergo structural, functional, and/or molecular plasticity (Undem and Taylor-Clark, 2014; Undem et al., 2015; Audrit et al., 2017; Verzele et al., 2021) and this is thought to be a factor contributing to disease morbidity. For example, biopsies taken from patients with chronic cough and asthma show an increased density of airway wall sensory nerve fibers (Ollerenshaw et al., 1991; O'Connell et al., 1995; Lee et al., 2003; Shapiro et al., 2021). In patients with chronic cough that is otherwise refractory to disease specific therapies, cough hypersensitivity syndrome is reduced when sensory neuron specific mechanisms are inhibited (Abdulqawi et al., 2015). Furthermore, in animal models of allergic asthma or respiratory viral infections, there is inflammatory cell influx into the vagal ganglia and the vagal sensory neurons themselves undergo transcriptional plasticity, especially with respect to genes associated with neurochemical phenotype and inflammatory signaling (Chuaychoo et al., 2005; Lieu et al., 2012; Zaccone et al., 2016; Verzele et al., 2021). Importantly, the accompanying airways hyperresponsiveness in these animal models is reduced following the selective inhibition of specific bronchopulmonary sensory neuron subtypes (Trankner et al., 2014; Reznikov et al., 2016). These observations suggest that the pathological events occurring during pulmonary inflammation adversely affect sensory neuron biology leading to the sensory manifestations of lung diseases (Mazzone and Undem, 2016). However, the mechanisms leading to these inflammation-evoked alterations in vagal sensory biology remain poorly described.

Many bronchopulmonary sensory neurons terminate in the airway mucosa in close apposition to the epithelium, mucosal glands, and resident or infiltrating immune cells (Mazzone et al., 2009; West et al., 2015; Shapiro et al., 2021). This spatial proximity makes it likely that chemical signaling axes exists between structural or inflammatory cells and bronchopulmonary sensory nerve terminals during times of inflammatory pathology. Several traditional candidate signaling molecules have been investigated for their potential to modulate vagal sensory neurons, including the prostaglandins, leukotrienes, and cytokines (Mazzone and Undem, 2016), although the clinical utility of targeting these pathways for alleviating pulmonary sensory neuron-dependent symptoms in patients has been underwhelming. More recently, interest has turned to several endogenous danger-signaling molecules because of their rapid release from stressed or infected cells and their ability to initiate and co-ordinate inflammation. One example is ATP, which is released from injured or infected epithelial cells and is a potent activator of subsets of vagal sensory neurons (Kwong et al., 2008; Lynch et al., 2020). Indeed, clinical trials with sensory neuron specific ATP receptor (P2X3) blockers are proving beneficial in some patients with chronic cough (Abdulqawi et al., 2015), evidence that targeting danger signaling pathways is clinically beneficial. Another molecule notably involved in orchestrating pulmonary inflammation is the tissue alarmin high mobility group box-1 (HMGB1). HMGB1 is a DNA binding protein that can translocate from the nuclear compartment into the cytoplasm when cells are injured, making it available for extracellular release (Di Candia et al., 2017; Imbalzano et al., 2017). Pathogens, including respiratory viruses, are particularly effective at triggering the release of HMGB1 from airway epithelial cells (Ullah et al., 2014; Hosakote et al., 2016; Arikkatt et al., 2017; Simpson et al., 2020), which promotes aspects of inflammation including smooth muscle proliferation and inflammatory cell recruitment. HMGB1 produces these proinflammatory effects through agonist activity at innate immune receptors, including the Receptor for Advanced Glycation End products (RAGE) and Toll-like Receptor 4 (TLR4), with the degree of receptor specificity depending on the oxidative state of the ligand (Janko et al., 2014; Yamasoba et al., 2016). For these reasons, HMGB1 has received significant recent attention as a targetable molecule for the treatment for asthma and related diseases (Imbalzano et al., 2017).

High mobility group box-1 was originally identified as amphorterin, a molecule shown to be instrumental in neuronal growth and development, and both RAGE and TLR4 are widely expressed in the nervous system, including sensory neurons (Hori et al., 1995; Allette et al., 2014). Therefore, in the present study, we set out to investigate whether HMGB1 can regulate the growth and/or function of vagal sensory neurons and to determine mechanisms involved.

## Materials and Methods

Experiments using specific pathogen-free wildtype (C57BL/6), RAGE deficient (RAGE^−/−^) and RAGE/TLR4 double deficient (RAGE^−/−^/TLR4^−/−^) mice were approved by an accredited institutional animal ethics committee in accordance with the Australian code for the care and use of animals for scientific purposes. Both males and females were used in approximately equal ratios, and as there were no noticeable sex-related differences, data were pooled for analyses. All mice used were adult (6–10weeks), except for mice inoculated with pneumovirus which were 7–14days old, as the role of epithelial-derived HMGB1 in viral pathogenesis is well-established in this model (Simpson et al., 2020).

### Recovery Procedures Under General Anesthesia

Mice underwent isoflurane general anesthesia and recovery for intratracheal delivery of the retrograde neuronal tracer cholera toxin B or for intranasal inoculation with virus or viral mimetics. Mice were anesthetized with isoflurane (4% induction, 1.25–1.5% maintenance) and procedures began once their withdrawal and palpebral reflexes had disappeared. For intratracheal injections (Mazzone et al., 2020; Verzele et al., 2021), their extrathoracic trachea was surgically exposed and 10μl of the retrograde tracer cholera toxin subunit b (recombinant), Alexa Fluor 488 conjugate (CTB-488; 0.5% in sterile saline; Thermo Fisher Scientific, Australia) was injected slowly (over 2min) into the tracheal lumen two cartilage rings below the larynx *via* a 10μl Hamilton syringe connected to a 30G needle. The needle was left in place for 30s to prevent backflow, following which the wound was sutured and animals recovered for 2weeks during which time body weight was monitored and post-operative analgesia was administered (Meloxicam, 1mg/kg, s.c for 48h). For intranasal delivery of virus or viral mimetics, mice were placed in an inclined position and 50μl of the viral mimetic polyI:C, 5 plaque-forming units (PFU) of mouse specific pneumovirus (J3666 strain), 10^5^ PFU of the A/Puerto Rico/8/1934 H1N1 (PR8) influenza A virus (IAV), or phosphate buffered saline (PBS; vehicle/mock controls) were administered into the nasal cavity, as previously described (Davidson et al., 2011; Verzele et al., 2021). Mice were allowed to recover from anesthesia and returned to their home cages for up to 7days post-inoculation (dpi) prior to tissue collection as described below.

### Vagal Ganglia Cell Culture

Vagal ganglia were harvested post-mortem from adult or juvenile mice euthanized with an overdose (100mg/kg, i.p.) of sodium pentobarbital and exsanguinated. Bilateral vagus nerves and ganglia (nodose-jugular complex) were excised and placed into sterile dishes containing modified Tyrodes solution (130mM NaCl, 20mM NaHCO_3_, 10mM HEPES, 3mM KCl, 10mM Glucose, 4mM CaCl_2_, 1mM MgCl_2_, and 0.5% antibiotic/antimitotic solution comprising penicillin, streptomycin, and amphotericin B), bubbled in 95% O_2_ and 5% CO_2_. Excess nerve and connective tissues were removed and ganglia neurons enzymatically dissociated using Trypsin/EDTA (250mg/ml) and Collagenase (1.33mg/ml) at 37°C for 1h as previously described (Kalous and Keast, 2010; McGovern et al., 2015). Enzyme-treated ganglia were washed and gently triturated to mechanically disperse the cells. Vagal sensory neurons were then harvested by centrifugation, resuspended in Neurobasal A medium containing B27 supplement, Glutamax I, and antibiotic/mycotic solution (Thermo Fisher Scientific; Australia), and transferred onto the center of glass coverslips pre-treated with Poly-L-lysine (5μg/ml; Sigma Aldrich, Australia) and Laminin (5μg/ml; Thermo Fisher Scientific, Australia). Coverslips were flooded with extra medium after 30–45min incubation at 37°C with 5% CO_2_, and the medium was replaced after 16–24h after which neurons were fed daily by removing and replacing 50% of the culture medium. Cultures were maintained for up to 4days and used for neurite outgrowth assays or electrophysiological recordings.

### Neurite Outgrowth Assays

Experiments were designed to assess the effect of HMGB1 on sensory neuron neurite outgrowth *in vitro*. Duplicate coverslips (technical replicates) were prepared with neurons dissociated from pooled left and right vagal ganglia obtained from individual adult mice, and this was repeated in 4–6 separate culture experiments (*n*=4–6). Neurons were cultured for 4days in medium that contained HMGB1 (100ng/ml) or its vehicle (PBS), following which culture medium was removed and replaced with 4% PFA (15min), before washing in 0.1M PBS, and neurite staining with antisera against β-tubulin III (see immunostaining section). The total number of neurons was counted for each coverslip to ensure comparable seeding densities, and five representative photomicrographs of fields of view (at ×100 magnification) per coverslip were taken for analysis. The number of cells with positive growth (defined as at least one neurite with a length greater than the diameter of the cell soma) was determined, following which all neurite projections arising from the soma of individual neurons were manually traced in ImageJ running the Neuron J plugin (Meijering et al., 2004), and the total neurite growth was recorded for each neuron in the field of view, with the average growth/neuron/coverslip calculated. The total neurite outgrowth for each individual experiment was expressed relative to the vehicle control outgrowth for that experiment. A minimum of five neurons per field of view were quantified. Average normalized total outgrowth was calculated for each condition and compared using an unpaired, two-tailed distributed, Welch’s *t*-test. Between group differences were considered statistically significant at *p*<0.05.

Additional experiments assessed the effect of prior *in vivo* pneumovirus infection on vagal sensory neuron neurite outgrowth *in vitro*. Duplicate coverslips (technical replicates) were prepared from neurons dissociated from pooled left and right vagal ganglia obtained from two mocks or pneumovirus infected juvenile mice (four ganglia in total), and this was repeated in 4–6 experiments (*n*=4–6). Neurons were cultured for 4days in the absence of any other treatments, following which culture medium was removed and replaced with 4% PFA (15min), before washing in 0.1M PBS, and neurite staining with antisera against β-tubulin III (see immunostaining section). Representative photomicrographs across the area of cell plating were taken with an Olympus BX-51 microscope equipped with an Olympus DP-72 camera, and the monolayer image files were loaded into StereoInvestigator 9.0 software (MBF Bioscience, Williston, VT, United States) for analysis. Because of the substantive growth observed in these experiments and the resultant inability to differentiate individual neurons from one another (see results), neurite length was estimated in an unbiased fashion using the Petrimetrics probe (with a Merz sine-wave radius of 25μm) in a similar manner to previous studies (Lipton et al., 2008). Briefly, a standard contour was created in which to place counting contours (frame size 50×50μm) over a 100×100mm grid. Any neurites intersecting the probe within the counting frame were scored. The total length of neurites calculated using the Petrimetrics probe was divided by the number of neuronal cell soma within each sample contour, to give the mean neurite length per cell. The observer who assessed neurite length was blind to the nature of all experimental groups. Average normalized total outgrowth was calculated for each condition and compared using a one-way ANOVA followed by a Tukey’s multiple comparisons *post-hoc* analysis. Between group differences were considered statistically significant at *p*<0.05.

### Whole Cell Patch Recordings of Dissociated Vagal Ganglia Neurons

Voltage- and current-clamp whole cell recordings were performed on dissociated vagal sensory neurons to measure HMGB1 evoked inward currents and changes in action potential firing rate. The patch pipettes were pulled on a P-87 microelectrode puller (Shutter Equipment Co., CA, United States) from borosilicate glass capillaries with an inner filament (Harvard Apparatus Ltd., Edenbridge, United Kingdom) and fire polished. When filled with pipette solution, the initial input resistance typically ranged between 3 and 6 MΩ. On the day of recording, coverslips with adherent cultured vagal sensory neurons were fixed inside of a custom fabricated recording chamber (volume~500μl), and the culture medium was replaced with bath solution at least 10min before recording. The pipette solution for voltage-clamp experiments was composed of (in mM): 126K-gluconate, 10 NaCl, 1 MgCl_2_, 10 EGTA, 2 NaATP, and 0.1 MgGTP, adjusted to pH 7.4 with KOH, osmolarity 290–300mOsm. Extracellular solution for voltage- and current-clamp experiments contained (in mM): 140 NaCl, 5 KCl, 2 CaCl_2_, 1 MgCl_2_, 10 HEPES, and 10 glucose, adjusted to pH 7.4 with NaOH, osmolarity 300–310mOsm. The pipette solution for current-clamp was composed of (in mM): 145K-gluconate, 2 MgCl_2_, 1 CaCl_2_, 10 EGTA, 5 HEPES, and 5 K_2_ATP, adjusted to pH 7.4 with KOH, osmolarity 290–300mOsm.

The recording chamber was mounted on the stage of an Olympus inverted microscope (New Hyde Park, United States) and continuously perfused with the extracellular bath solution (~2ml/min). The electrode was positioned on cell membranes of isolated neurons chosen at random using a micromanipulator (MP-285, Sutter Instrument Company, United States) and access to the cell interior was judged by the appearance of a transient membrane capacitance current after breaking the membrane by negative pressure through recording pipette (Axopatch-200A amplifier, Axon Instruments, United States). After obtaining a giga-ohm seal, the pipette potential was held at −60mV and voltage-steps (10mV, 200ms duration) were delivered periodically to monitor the capacitance and access resistance. Whole cell capacitance and series resistance (using only cells with <30 MΩ) were compensated (>80%) before experimentation, and leak subtraction was performed. The change in series resistance over the course of each experiment was also monitored, and recordings with a greater than 20% change in series resistance were excluded from the final data analysis. The electrical signals were low-pass-filtered at 2KHz and sampled at 1KHz in our recording protocols. Membrane current and voltage responses were determined during acute exposure to 200ng/ml recombinant HMGB1 (Sigma Aldrich, Australia) by addition to the ongoing perfusate across the cells. Data points presented in figures represent individual neuron responses obtained from successful recordings in 3–4 separate culture experiments.

### Immunostaining

Immunostaining of markers of interest was performed on 4% PFA fixed dissociated neurons grown on coverslips in culture or on slide mounted cryostat cut (12μm) sections of vagal ganglia, prepared from control (no intervention), mock or virus (pneumovirus or influenza A) exposed mice. In all cases, samples were blocked in 10% goat serum in PBS for 1h before incubation with primary antibodies for chicken anti-beta-tubulin III, (Abcam #41489; 1:600 room temperature for 2h) to label neurites of cultured neurons or rabbit anti-HMGB1 (Abcam, #18256; 1:1000, room temperature overnight) to stain for nuclear and cytoplasmic HMGB1 in tissue sections. Samples were subsequently washed before incubation with secondary antibody (goat anti-chicken or anti-rabbit Alexa Fluor 594 or 488, 1:500, Thermo Fisher Scientific, Australia) for 1h at room temperature. Antibodies were diluted in PBS containing 2% goat serum and 0.3% triton X100. Coverslips were mounted onto glass microscope slides with Prolong Gold antifade reagent containing DAPI (Thermo Fisher Scientific, Australia) and viewed with an Olympus BX-51 microscope equipped with an Olympus DP-72 camera. Neurite outgrowth in cultured neurons was quantified as described above. Images were taken with Leica DFC7000 T camera (100x magnification) at the same exposure time for offline analysis (Image J) of HMGB1 nucleus-to-cytoplasm translocation on slide mounted tissue sections. Images were converted to 8-bit greyscale and for every neuron with an identified nucleus (DAPI), the intensity of HMGB1 staining was determined within the nucleus and cytoplasm by determining the mean grey value of each. If the value was higher in the nucleus then this was counted as positive nuclear staining and if the value was higher in the cytoplasm, then this counted as cytoplasmic translocation. This was performed for all neurons with a visible nucleus across all vagal ganglia sections and the percentage of HMGB1 cytoplasmic translocation was calculated from the total number of neurons counted divided by the number of neurons that had undergone cytoplasmic translocation. Mean±SEM was calculated for all groups and comparisons made using an unpaired, two-tailed distributed, Welch’s *t* test. Between group differences were considered statistically significant at *p*<0.05.

### Single Cell RNA Analyses

We have previously reported a transcriptomic analysis of individual murine bronchopulmonary vagal sensory neurons (Mazzone et al., 2020). In the present study, we undertook a reanalysis of this single cell RNAseq dataset to specifically investigate the transcriptional expression of *Hmgb1*, *Ager* (RAGE), and *Tlr4* in vagal sensory neurons innervating the airways and lungs. As previously described (Mazzone et al., 2020), we used the relative expression of the P2X2 purinergic receptor gene (*P2rx2*) as a surrogate marker for jugular (*P2rx2*^LOW^) and nodose (*P2rx2*^HIGH^) neurons (Kwong et al., 2008) using an (unbiased) two component gaussian mixture model analysis on log normalized *P2rx2* cell expression counts to separate cells into these two clusters. Expressions levels of *Hmgb1*, *Ager*, and *Tlr4* within each cluster were visualized with violin plots and average group expressions compared (Mazzone et al., 2020). Statistical associations between *Ager* and *Tlr4* expression levels and common marker genes that define sensory neuron subtypes (*Trpv1*, *Trpa1*, *Calca*, and *Tac1*) were also investigated in individual neurons. Gene expression values are quoted as the log2 counts per million (CPM), and sequencing data are deposited in the GEO repository, accession number GSE161878.

In subsequent experiments, we used RNAscope to validate single cell RNAseq gene expression patterns in vagal bronchopulmonary sensory neurons. Animals previously receiving an intratracheal injection of CTB-488 were euthanized with an overdose of sodium pentobarbital (100mg/kg, i.p.) and transcardially perfused with DEPC-treated PBS, followed by 4% of DEPC-treated PFA. Vagal ganglia were removed, processed and 10μm sections were collected on a cryostat (−20°C), sequentially over four slides (Superfrost plus) for each pair of vagal ganglia from one animal. A minimum of four slides from four different animals were processed for each probe set. Sections were pretreated in 0.3% hydrogen peroxide solution and incubated at room temperature for 10min, then washed twice with distilled water. Sections were immersed in RNAscope target retrieval buffer (ACD, 322000), maintained at 92–94°C for 15min. Slides were removed, washed twice in distilled water, and then rinsed with 100% ethanol and allowed to dry. A hydrophobic barrier was created around the sections and air dried for 5min. Protease plus (ACD, 322281) was added to the samples and incubated in a HybEZ oven (ACD, 321461) for 30min at 40°C. After incubation, sections were washed twice in distilled water. Hybridization and amplification steps were performed following the RNAscope protocol provided by ACD. Probes were hybridized for 2h at 40°C in a HybEZ oven. The following probes were multiplexed in combination with Mm-*P2rx2* (ACD, ADV443681) and either Mm-*Ager* (ACD, ADV550791) or Mm-*Tlr4* (ACD, ADV316801). Slides were washed with 1X wash buffer (ACD, 310091), twice for 2min each at room temperature following each amplification step using RNAscope multiplex fluorescent assay v2 (ACD, 323100). Detection of *P2rx2* was completed using Opal-Cy3 (1:1,000; Perkin Elmer, 745E001KT), and other genes of interest were detected using Opal-Cy5 (1:1,000; Perkin Elmer, 744001KT) for 30min at 40°C. Slides were washed twice for 2min in 1X wash buffer, rinsed with distilled water, and coverslipped with Prolong gold antifade reagent with DAPI. A positive and negative probe set (ACD ADV320881, ACD and ADV320871, respectively) were used to verify high positive control signals and no negative levels of background noise. Sections were visualized on a fluorescent microscope (Leica DM6 B) with all CTB-488 positive neurons (bronchopulmonary vagal sensory neurons), along with untraced neurons and assessed for expression of each gene of interest described above. Images were taken (200x magnification) at the same exposure time for each gene across each sample for offline analysis. Given the complete absence of any non-specific signal in negative probe control tissue sections, the expression of a gene was considered present if positive probe tissues showed one or more probe count (pixel) present within each neuron. The number of neurons expressing each gene in both CTB-488 traced and untraced neurons were counted and expressed as the percentage of the total number of neurons counted across all vagal ganglia sections for one animal. Mean±SEM was calculated for each group and compared using a one-way ANOVA followed by a Tukey’s multiple comparisons *post-hoc* analysis. Between group differences were considered statistically significant at *p*<0.05.

## Results

### Expression of HMGB1, RAGE, and TLR4 in Vagal Sensory Neurons

**Figure 1:**
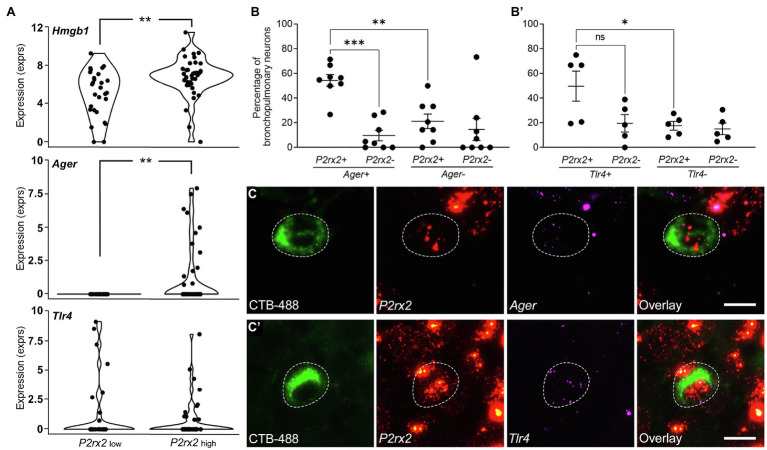
Gene expression of *Hmgb1*, *Ager* (RAGE), and *Tlr4* in bronchopulmonary vagal sensory neurons clustered by *P2rx2* expression levels. **(A)** Violin plots of single cell RNAseq data showing the mean log_2_CPM values for *P2rx2* and *Hmgb1*, *Ager*, or *Tlr4* genes correlated to *P2rx2* expression. *P2rx2^LOW^* and *P2rx2*^HIGH^ are presumed jugular-like and nodose-like bronchopulmonary vagal sensory neurons, respectively (Mazzone et al., 2020). ^**^p<0.01 significantly different mean expression levels. RNAscope was performed on bronchopulmonary vagal sensory neurons for *Ager* **(B)** or *Tlr4* **(B’)** and quantified relative to percentage of neurons showing a presence or absence of *P2rx2* expression. Representative photomicrographs demonstrate bronchopulmonary neurons (retrogradely traced with CTB-488, green) and the expression of *P2rx2* (red) and either **(C)** *Ager* or **(C’)** *Tlr4* (magenta). Neuronal soma outlined with dashed lines. Scale bar equals 20μm. ^*^p<0.05; ^**^p<0.01; and ^***^p<0.001 significantly different expression levels as determined by multiple comparisons, two-way ANOVA. ns, not significant.

Reanalysis of our existing single cell RNAseq library (Mazzone et al., 2020) revealed transcriptional expression of *Hmgb1*, *Ager*, and *Tlr4* in identified bronchopulmonary vagal sensory neurons. About 95.5% of the neurons contained *Hmgb1* transcripts, whereas *Ager* and *Tlr4* transcripts were detected in approximately 19.4 and 28.3% of neurons, respectively (Figure 1A). Interestingly *Ager* expression (but not *Tlr4*) was restricted to neurons with high *P2rx2* expression (presumed nodose neurons (Kwong et al., 2008), although the actual expression levels of *Ager* and *P2rx2* were not correlated (FDR=0.752). However, *Ager* expression was highly correlated (FDR=6.2×10^−3^) with the nociceptor marker *Trpv1* while *Tlr4* expression correlated (FDR=0.013) with *Calca*, a marker of a subset of peptidergic neurons. No relationships were seen for *Ager* or *Tlr4* with *Trpa1* or *Tac1* (FDR=0.8–1.0). Only one neuron expressed detectable transcripts for both *Ager* and *Tlr4*, suggestive of largely different neuron subpopulations expressing these receptors for HMGB1.

The expression of *Ager* and *Tlr4* in bronchopulmonary vagal ganglia neurons was confirmed by RNAscope (Figures 1B,C). However, the results of this method revealed a higher percentage of bronchopulmonary neurons expressing *Ager* (60.1%) and *Tlr4* (67.2%), perhaps reflecting an increased sensitivity of the assay given our relatively low inclusion level for positive marker expression (one dot per cell). Approximately 54.4% of *P2rx2* expressing bronchopulmonary traced neurons co-expressed *Ager* (Figure 1B), while 49.7% co-expressed *Tlr4* (Figure 1B’). Similar to the RNAseq data, *Ager* (but not *Tlr4*) expression was significantly less frequent in neurons devoid of *P2rx2* expression, indicative of enrichment of *Ager* in bronchopulmonary nodose neurons. Neurons that were not retrogradely traced from the airways and lungs (presumed to mostly represent vagal sensory neurons innervating other tissues) also expressed *Ager* (43.5% of neurons) and *Tlr4* (56.4% of neurons). Interestingly, the percentage of untraced (~28.7%) and traced (~54.4%) *P2rx2* expressing (nodose) neurons that also expressed *Ager* was significantly different (*p*<0.0304), suggesting that bronchopulmonary nodose neurons more frequently express *Ager* relative to the general nodose population. Collectively, these RNAseq and RNAscope analyses suggest a sizable proportion of vagal ganglia neurons are conceivably responsive to HMGB1 either *via* RAGE or TLR4 expression.

### Functional Responses to HMGB1 in Vagal Sensory Neurons

**Figure 2:**
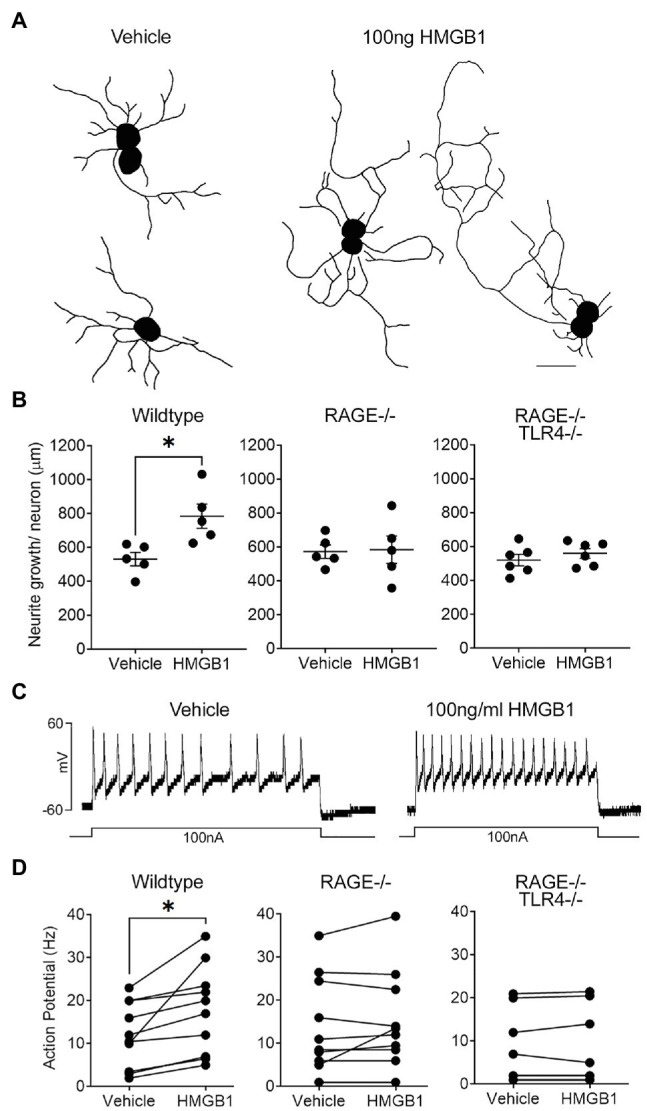
High mobility group box-1 (HMGB1) evokes Receptor for Advanced Glycation End products (RAGE)-dependent neurite outgrowth and alters the electrophysiological properties of vagal sensory neurons. **(A)** Representative individual neurite outgrowth tracings of vagal sensory neurons cultured in the presence of vehicle and HMGB1. Scale bar=50μm. **(B)** Quantitative data showing the effects of HMGB1 on neurite outgrowth in neurons from wildtype, RAGE deficient (RAGE^−/−^), and RAGE/Toll-like Receptor 4 (TLR4) double deficient (RAGE^−/−^/TLR^−/−^) mice. Each dot represents one animal with the average neurite outgrowth of a minimum of five cells manually traced and analyzed for one replicate coverslip (*n*=5 experiments) with overall group mean and SEM plotted. **(C)** Representative current clamp recordings from a wild-type vagal ganglia neuron showing injected current evoked action potential formation (spiking) in the presence of vehicle and HMGB1. **(D)** Quantitative data showing action potential firing frequency (spikes per second, Hz) for individual neurons obtained from wildtype, RAGE^−/−^, and RAGE^−/−^/TLR^−/−^ mice in the presence of vehicle or HMBG1. ^*^p<0.05 significantly different to vehicle as determined by unpaired *t* test.

Dissociated vagal sensory neurons from adult mice, regardless of genotype, progressively grew neurites over the course of 4days in culture, after which 85–90% of neurons on each coverslip possessed at least one neurite with a length greater than the diameter of the cell soma (i.e., positive growth). On average, neurons from adult wildtype, RAGE^−/−^ and RAGE^−/−^/TLR4^−/−^ mice in vehicle-treated cultures possessed neurites of comparable total lengths (587±83, 573±32, and 520±37μm/cell, respectively; Figure 2). Treatment of cultured neurons from wildtype mice with HMGB1 resulted in a significant increase in neurite outgrowth equivalent to approximately 141% of that in vehicle-treated controls (Figure 2B) but did not change the percentage of neurons exhibiting positive growth (85–90% per coverslip). By contrast, HMGB1 was without effect on neurite outgrowth in all but one experiment using RAGE^−/−^ neuron cultures and abolished in all experiments using RAGE^−/−^/TLR4^−/−^ neuron cultures (Figure 2B).

In electrophysiological studies, bath application of HMGB1 evoked a measurable (greater than 10pA) inward current in 70% of wildtype neurons tested (average response=−40.0±30.9 pA; range=−13 to −335 pA; and *n*=10). Furthermore, action potential induction in response to a 100nA current injection was significantly enhanced by HMGB1 (17.8±3.2Hz) compared to vehicle (12.0±2.4Hz), although this effect was more robust in some cells compared to others (Figures 2C,D). By contrast, HMGB1-evoked responses were present in only 11 percent of neurons isolated from RAGE^−/−^ animals. Thus, measurable inward currents in response to HMGB1 were absent in nine out of 12 cells examined and ranged from −20.5 to −247.1pA in the remaining three cells (overall mean HMGB1-evoked response=−26.5±22.3pA, not significantly different to vehicle). Action potential induction was similarly not altered by HMGB1 in nine of the 11 cells examined from RAGE^−/−^ animals (Figure 2D). None of the examined neurons harvested from RAGE^−/−^/TLR4^−/−^ mice demonstrated inward current or enhanced action potential responses to bath application of HMGB1 (Figure 2D).

### Effects of Respiratory Viruses on Neurite Outgrowth and Neuronal HMGB1

**Figure 3:**
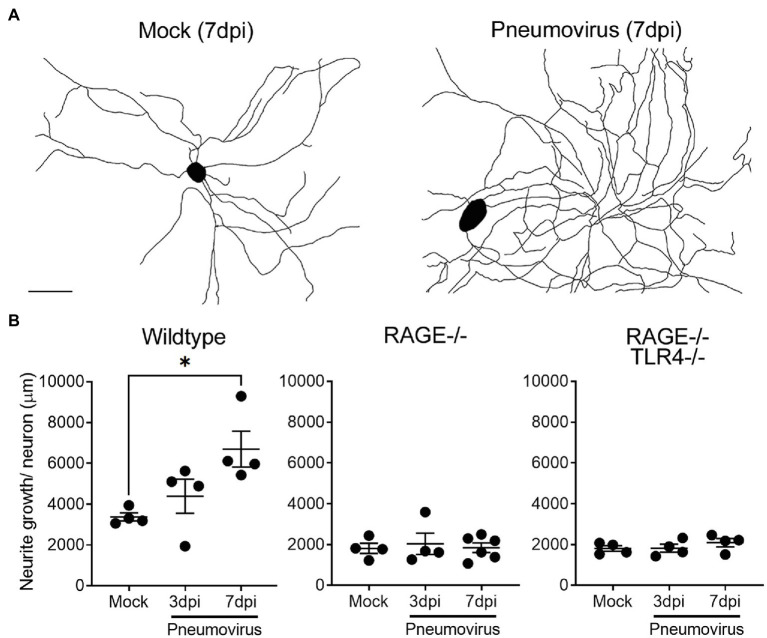
*In vivo* respiratory pneumovirus infection evokes RAGE-dependent upregulation of vagal sensory neurite outgrowth. **(A)** Representative neurite outgrowth tracings of cultured vagal ganglia neurons harvested 7days post-inoculation (7dpi) *in vivo* with vehicle or five plaque forming units of pneumovirus (PVM). Scale bar=50μm. **(B)** Quantitative data showing the time dependent (3–7dpi) effects of *in vivo* pneumovirus infection on neurite outgrowth in neurons cultured from wildtype, RAGE deficient (RAGE^−/−^), and RAGE/TLR4 double deficient (RAGE^−/−^/TLR4^−/−^) mice. Each dot represents the average neurite outgrowth per cell estimated with an unbiased Petrimetircs probe covering within a 100×100mm grid from one replicate coverslip (*n*=4–6 experiments) with overall group mean and SEM plotted. ^*^p<0.05 significantly different to 7dpi mock as determined by one-way ANOVA.

After 4days in culture, neurite outgrowth in dissociated vagal ganglia neurons prepared from juvenile animals was consistently greater than that observed with comparable cultures prepared from adult animals (compare mock treated growth in Figures 2A, 3A). Neurite outgrowth exhibited a further significant upregulation in cultures prepared from ganglia obtained from mice 7days following intranasal infection with pneumovirus (Figure 3). Thus, compared to neurons cultured from mock control animals (also collected 7days after mock inoculation and cultured for a further 4days), neurons from pneumovirus-infected animals displayed ~twice the average neurite outgrowth (Figure 3B). Interestingly, unlike in cultures of adult neurons (see Figure 2), wildtype neurons from juvenile animals displayed significantly (*p*<0.01) more average basal neurite outgrowth in culture (3,436±160μm) when compared to equivalent cultures of RAGE^−/−^ and RAGE^−/−^/TLR4^−/−^ neurons (1813±176 and 1808±134μm, respectively). Strikingly, the upregulation of neurite outgrowth seen in neurons from pneumovirus infected wildtype animals was absent in neurons cultured from pneumovirus infected RAGE^−/−^ and RAGE^−/−^/TLR4^−/−^ animals (Figure 3B).

**Figure 4:**
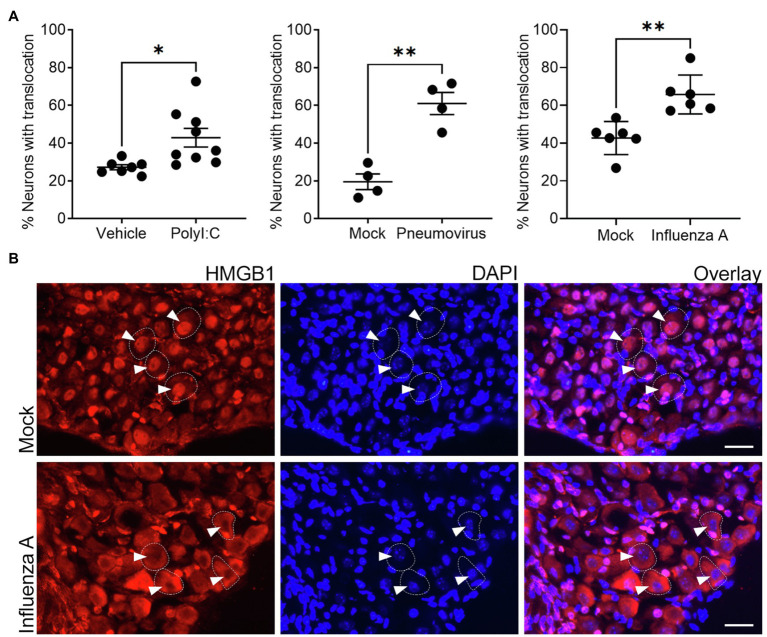
*In vivo* exposure to viral mimetic or respiratory viral infection evokes nuclear to cytoplasm translocation of HMGB1 in vagal sensory neurons. **(A)** Significantly more frequent cytoplasmic HMGB1 staining was observed in vagal sensory neurons following *in vivo* inoculation with either the viral mimetic PolyI:C or viral infection with murine pneumovirus and influenza A virus (IAV) in wildtype mice. The quantitative data represent the percentage of neurons with detectable cytoplasmic HMGB1 staining. Each dot represents the average of 825±154 neurons per animal with the overall group mean and SEM plotted. **(B)** Representative photomicrographs show examples of the redistribution of HMGB1 staining in vagal sensory neurons following influenza A respiratory viral infection. Vagal sensory ganglia were collected at 7dpi for all conditions and tissue sections stained for HMGB1 and the nuclear marker DAPI. Dashed outlines indicate example neurons. Arrowheads point to neuronal nuclei (as determined by DAPI stain). ^*^p<0.05 and ^**^p<0.01 significantly different to time-matched vehicle/mock control as determined by unpaired *t* test scale bar=25μm.

The RAGE-dependent upregulation of neurite outgrowth following prior *in vivo* pneumovirus infection suggests a role for HMGB1 in respiratory viral induced upregulation of neurite outgrowth. We have previously reported epithelial release of HMGB1 following respiratory virus infection or allergic inflammation in mice using this same experimental model (Ullah et al., 2014; Simpson et al., 2020). However, our transcriptomic data (Figure 1) suggest that another potential source of HMGB1 is the bronchopulmonary vagal sensory neurons themselves. Consistent with this, in control animals given an intranasal treatment with vehicle (PBS), HMGB1 was notably confined to the nucleus of the majority of vagal ganglia sensory neurons. By contrast, significantly more neurons displayed cytoplasmic HMGB1 expression following intranasal exposure to the viral mimetic PolyI:C (Figure 4A). This mobilization of HMGB1 into the cytoplasmic compartment of vagal sensory neurons following viral mimetic exposure was also evident with two different active respiratory viral infections. Thus, the number of vagal sensory neurons displaying cytoplasmic HMGB1 protein was also significantly increased (compared to mock-inoculated controls) by both intranasal pneumovirus inoculation (7dpi in juvenile mice) and intranasal IAV inoculation (7dpi in adult mice; Figures 4A,B).

## Discussion

High mobility group box-1 is present in the nucleus of most cells where it functions as a nonhistone DNA binding protein. However, HMGB1 also plays a role in inflammation, growth, and repair, acting as an early alarmin when released from activated, injured, or necrotic cells. This action requires translocation of HMGB1 from the nucleus into the cytoplasm making it available for extracellular release (Hosakote et al., 2016; Arikkatt et al., 2017; Di Candia et al., 2017; Imbalzano et al., 2017; Parker et al., 2017). The extracellular biological actions of HMGB1 occur largely *via* RAGE and TLR4, dependent upon the oxidative state of the ligand. Thus, all-thiol HMGB1 acts through RAGE and disufide HMGB1 acts through TLR4 (Janko et al., 2014; Yamasoba et al., 2016; Ma et al., 2017). In pulmonary disease, especially pathologies involving pathogen exposure, respiratory epithelial cells, and infiltrating leukocytes release large amounts of HMGB1, which acts as an early regulator of innate immunity and inflammation (Hosakote et al., 2016; Arikkatt et al., 2017; Di Candia et al., 2017; Imbalzano et al., 2017). Our data argue that vagal sensory neurons innervating the airways and other tissues have the capacity to detect and/or release HMGB1, and this may have important implications in the development of sensory hyperinnervation and hypersensitivity in viral respiratory disease.

Previous studies have shown that HMGB1 can promote neuronal growth (Saleh et al., 2013; Merianda et al., 2015) and evoke Ca^2+^ influx, inward currents, and enhanced action potential firing in dissociated dorsal root ganglia sensory neurons (Feldman et al., 2012), notably *via* the activation of RAGE. Similarly, HMGB1 acting *via* RAGE on dorsal root ganglia sensory neurons is involved in the development of sensitization and mechanical hyperalgesia in models of neuropathic pain (Allette et al., 2014), and antagonism of RAGE has been shown to suppress peripheral axon regeneration after nerve injury (Rong et al., 2004). These published data argue that the all-thiol form of HMGB1 (and hence RAGE) plays a predominant role in modulating sensory neurons, largely consistent with findings in the present study. Indeed, our transcriptional analyses confirm the expression of both RAGE and TLR4 in subsets of vagal sensory neurons displaying a nociceptor phenotype (i.e., neurons expressing TRPV1 or the neuropeptide CGRP), while our functional studies show that HMGB1 both excites and enhances the growth of vagal ganglia sensory neurons, responses that were largely abrogated in neurons lacking RAGE expression. However, a very recent study suggests TLR4 expression in DRG neurons may be important in nerve injury-mediated hypersensitivity specifically in female mice (Szabo-Pardi et al., 2021), something that our study did not address.

Sensory hypersensitivity is a common feature of pulmonary disease. Structural and immune cells in the airways and lungs represent potential sources of HMGB1, which can be released extracellularly in the diseased airways. Accordingly, this pulmonary cellular supply of HMGB1 would then be available to act on nearby sensory nerve terminals that are juxtaposed to release sites, because many vagal sensory neurons express RAGE. This may represent an important early mechanism that enhances sensory neural responses needed for pulmonary defense during times of “danger signaling.” Intriguingly, our data point to possible enrichment of RAGE in bronchopulmonary neurons, as retrogradely traced neurons showed higher *Ager* expression than did untraced neurons. This observation warrants confirmation in carefully designed studies assessing tissue specific and well-characterized vagal ganglia neurons, as it may suggest an especially important role for sensory neuron RAGE in pulmonary defense. Additionally, our findings argue that vagal sensory neurons themselves may represent an alternative source of HMGB1, mobilized within the neuron soma in the vagal ganglia, perhaps as a consequence of ongoing pulmonary inflammation or pathogen induced sensory neuron activation or damage. Our data support this suggestion, as we noted widespread sensory neuron cytoplasmic HMGB1 translocation following viral mimetic exposure or respiratory viral infections. Consistent with this, Yang and colleagues recently demonstrated that persistent optogenetic stimulation of somatic nociceptors results in HMGB1 nucleus to cytoplasm translocation, while genetic deletion of neuronal HMGB1 protected against the development of cutaneous inflammation and allodynia following peripheral nerve injury (Yang et al., 2021). Indeed, we have shown that our murine models of respiratory viral infection develop lung pathologies consistent with severe inflammatory disease, with bronchial, interstitial, alveolar, and vascular inflammation driven by lymphocytic inflammatory cell infiltrates and elevated lung interferons, TNFa, and other cytokines (Ullah et al., 2014; Arikkatt et al., 2017; Verzele et al., 2021). Under these circumstances, it is expected that persistent and strong stimuli for sensory neuron discharge exist, and perhaps this is the prerequisite for vagal ganglia neurons to mobilize HMGB1.

Neuropathic pain, resultant from nerve damage or diabetes, is similarly associated with translocation of HMGB1 in dorsal root ganglia sensory neurons (Shibasaki et al., 2010) where it becomes available for signaling at neighboring neurons, glia and resident cells within the sensory ganglia and surrounding nerve. A paracrine action might serve to propagate a wave of signaling in the ganglia, explaining why so many neurons demonstrate HMGB1 translocation in nerve injury models (Shibasaki et al., 2010) and in our models of lung pathology in the present study. This is not the first evidence we have presented for widespread neuroinflammatory events in the vagal ganglia following respiratory viral infections. We previously reported that influenza A respiratory virus infection promotes inflammatory cell recruitment to the vagal ganglia accompanied by an increase in the ganglionic expression of many inflammatory genes in vagal ganglia neurons (Verzele et al., 2021). In that study, we also noted that sensory ganglia unrelated to lung function (the lumbar DRG) did not show signs of inflammation, while vagal ganglia neurons innervating pulmonary and non-pulmonary tissues, such as the esophagus, both showed signs of neuroinflammatory changes. These data argue in favor of specific vagal signaling mechanisms, rather than humoral stimuli associated with systemic inflammation, underpinning the development of vagal ganglia inflammation. In light of the present data, it is tempting to speculate that neuronally released HMGB1 may represent an important ganglionic alarm signaling mechanism that initiates these neuroinflammatory events, *via* a paracrine action on neighboring neurons or glia and/or through inflammatory cell chemotaxis. Regardless, the present data provide further evidence to support our prior observation (Verzele et al., 2021) that vagal sensory neurons projecting to target tissues other than the pulmonary system may be impacted by pulmonary infection and inflammation, although the functional consequences of such an impact, and mechanisms underpinning it, requires further investigation.

The findings of the present study raise several important questions for future investigation. Firstly, given that RAGE activation is associated with increased vagal ganglia sensory neurite outgrowth *in vitro*, it is tempting to speculate that this may represent a mechanism involved in the development of bronchopulmonary hyperinnervation in patients with chronic lung inflammation (Ollerenshaw et al., 1991; O'Connell et al., 1995; Lee et al., 2003; Shapiro et al., 2021). Nevertheless, it remains to be seen whether sensory neuron RAGE activation *in vivo*, either by lung or ganglia derived HMGB1, is an effective stimulus for peripheral nerve terminal sprouting and branching, needed for bronchopulmonary innervation to increase. Unfortunately, our *in vivo* studies using severe viral infections are not suitable for assessing inflammatory-induced changes to bronchopulmonary innervation because of the short duration of the model. An alternative chronic model of lung inflammation is better suited to addressing this. It is also of interest to question the mechanisms underpinning the rapid electrophysiological effects evoked by HMGB1 activation of sensory neuron RAGE. The induction of inward currents and the altered rheobase leading to increased action potential spiking are consistent with RAGE activation acutely gating a yet to be identified sensory neuron ion channel. DRG sensory neurons express other innate immune receptors, including TLR7, and when activated induce rapid ionic currents due to the gating of sensory neuron TRP channels (Park et al., 2014). Indeed, RAGE activation is associated with enhanced TRPV1 channel gating in models of diabetic neuropathy (Lam et al., 2018), which could conceivably contribute to the electrophysiological effects of HMGB1 in the present study. Lastly, whether the vagal source of HMGB1 is an important regulator of pulmonary inflammation remains to be tested, but as shown recently by Yang et al., 2021 neuronal HMGB1 subserves this function in other models of inflammation.

In conclusion, these findings suggest a novel mechanism of vagal sensory nerve dysfunction and plasticity secondary to tissue alarmin release, highlighting possible therapeutic avenues to explore targeting the HMGB1-RAGE signaling axis in patients with respiratory disease.

## Data Availability Statement

The datasets presented in this study can be found in online repositories. The names of the repository/repositories and accession number(s) can be found at: GEO, GSE161878.

## Ethics Statement

The animal study was reviewed and approved by The University of Melbourne or University of Queensland Animal Ethics Committees.

## Author Contributions

SM, AEM, KS, and SP: conception and design. S-KY, JK, JS, JA, MF, AAM, CC, LT, MR, BC, and AEM: data generation and analysis. SM and AEM: drafting of the paper. All authors contributed to the article and approved the submitted version.

## Funding

This work was supported by grants and fellowship awards to SM, AEM, and KS from the National Health and Medical Research Council of Australia (APP1078943, APP1025589, and APP1161029).

## Conflict of Interest

The authors declare that the research was conducted in the absence of any commercial or financial relationships that could be construed as a potential conflict of interest.

## Publisher’s Note

All claims expressed in this article are solely those of the authors and do not necessarily represent those of their affiliated organizations, or those of the publisher, the editors and the reviewers. Any product that may be evaluated in this article, or claim that may be made by its manufacturer, is not guaranteed or endorsed by the publisher.

## References

[ref1] Abdulqawi R. Dockry R. Holt K. Layton G. McCarthy B. G. Ford A. P.. (2015). P2X3 receptor antagonist (AF-219) in refractory chronic cough: a randomised, double-blind, placebo-controlled phase 2 study. Lancet 385, 1198–1205. doi: 10.1016/S0140-6736(14)61255-1, PMID: 25467586

[ref2] Allette Y. M. Due M. R. Wilson S. M. Feldman P. Ripsch M. S. Khanna R.. (2014). Identification of a functional interaction of HMGB1 with receptor for advanced glycation end-products in a model of neuropathic pain. Brain Behav. Immun. 42, 169–177. doi: 10.1016/j.bbi.2014.06.199, PMID: 25014009 PMC4560334

[ref3] Arikkatt J. Ullah M. A. Short K. R. Zhang V. Gan W. J. Loh Z.. (2017). RAGE deficiency predisposes mice to virus-induced paucigranulocytic asthma. elife 6:e21199. doi: 10.7554/eLife.21199, PMID: 28099113 PMC5243115

[ref4] Audrit K. J. Delventhal L. Aydin O. Nassenstein C. (2017). The nervous system of airways and its remodeling in inflammatory lung diseases. Cell Tissue Res. 367, 571–590. doi: 10.1007/s00441-016-2559-7, PMID: 28091773

[ref5] Chuaychoo B. Hunter D. D. Myers A. C. Kollarik M. Undem B. J. (2005). Allergen-induced substance P synthesis in large-diameter sensory neurons innervating the lungs. J. Allergy Clin. Immunol. 116, 325–331. doi: 10.1016/j.jaci.2005.04.005, PMID: 16083787

[ref6] Davidson S. Kaiko G. Loh Z. Lalwani A. Zhang V. Spann K.. (2011). Plasmacytoid dendritic cells promote host defense against acute pneumovirus infection via the TLR7-MyD88-dependent signaling pathway. J. Immunol. 186, 5938–5948. doi: 10.4049/jimmunol.1002635, PMID: 21482736 PMC3404606

[ref7] Di Candia L. Gomez E. Venereau E. Chachi L. Kaur D. Bianchi M. E.. (2017). HMGB1 is upregulated in the airways in asthma and potentiates airway smooth muscle contraction via TLR4. J. Allergy Clin. Immunol. 140, 584–587.e8. doi: 10.1016/j.jaci.2016.11.049, PMID: 28259445 PMC5540224

[ref8] Feldman P. Due M. R. Ripsch M. S. Khanna R. White F. A. (2012). The persistent release of HMGB1 contributes to tactile hyperalgesia in a rodent model of neuropathic pain. J. Neuroinflammation 9:180. doi: 10.1186/1742-2094-9-180, PMID: 22824385 PMC3488576

[ref9] Hori O. Brett J. Slattery T. Cao R. Zhang J. Chen J. X.. (1995). The receptor for advanced glycation end products (RAGE) is a cellular binding site for amphoterin. Mediation of neurite outgrowth and co-expression of rage and amphoterin in the developing nervous system. J. Biol. Chem. 270, 25752–25761. doi: 10.1074/jbc.270.43.25752, PMID: 7592757

[ref10] Hosakote Y. M. Brasier A. R. Casola A. Garofalo R. P. Kurosky A. (2016). Respiratory syncytial virus infection triggers epithelial HMGB1 release as a damage-associated molecular pattern promoting a monocytic inflammatory response. J. Virol. 90, 9618–9631. doi: 10.1128/JVI.01279-16, PMID: 27535058 PMC5068515

[ref11] Imbalzano E. Quartuccio S. Di Salvo E. Crea T. Casciaro M. Gangemi S. (2017). Association between HMGB1 and asthma: a literature review. Clin. Mol. Allergy 15:12. doi: 10.1186/s12948-017-0068-1, PMID: 28630596 PMC5471678

[ref12] Janko C. Filipovic M. Munoz L. E. Schorn C. Schett G. Ivanovic-Burmazovic I.. (2014). Redox modulation of HMGB1-related signaling. Antioxid. Redox Signal. 20, 1075–1085. doi: 10.1089/ars.2013.5179, PMID: 23373897 PMC3928832

[ref13] Kalous A. Keast J. R. (2010). Conditioning lesions enhance growth state only in sensory neurons lacking calcitonin gene-related peptide and isolectin B4-binding. Neuroscience 166, 107–121. doi: 10.1016/j.neuroscience.2009.12.019, PMID: 20006678

[ref14] Kwong K. Kollarik M. Nassenstein C. Ru F. Undem B. J. (2008). P2X2 receptors differentiate placodal vs. neural crest C-fiber phenotypes innervating Guinea pig lungs and esophagus. Am. J. Phys. Lung Cell. Mol. Phys. 295, L858–L865. doi: 10.1152/ajplung.90360.2008, PMID: 18689601 PMC2584877

[ref15] Lam D. Momeni Z. Theaker M. Jagadeeshan S. Yamamoto Y. Ianowski J. P.. (2018). RAGE-dependent potentiation of TRPV1 currents in sensory neurons exposed to high glucose. PLoS One 13:e0193312. doi: 10.1371/journal.pone.0193312, PMID: 29474476 PMC5825096

[ref16] Lee S. Y. Kim M. K. Shin C. Shim J. J. Kim H. K. Kang K. H.. (2003). Substance P-immunoreactive nerves in endobronchial biopsies in cough-variant asthma and classic asthma. Respiration 70, 49–53. doi: 10.1159/000068413, PMID: 12584391

[ref17] Lieu T. M. Myers A. C. Meeker S. Undem B. J. (2012). TRPV1 induction in airway vagal low-threshold mechanosensory neurons by allergen challenge and neurotrophic factors. Am. J. Phys. Lung Cell. Mol. Phys. 302, L941–L948. doi: 10.1152/ajplung.00366.2011, PMID: 22345578 PMC3362153

[ref18] Lipton J. W. Tolod E. G. Thompson V. B. Pei L. Paumier K. L. Terpstra B. T.. (2008). 3,4-Methylenedioxy-N-methamphetamine (ecstasy) promotes the survival of fetal dopamine neurons in culture. Neuropharmacology 55, 851–859. doi: 10.1016/j.neuropharm.2008.06.062, PMID: 18655796 PMC2572681

[ref19] Lynch J. P. Werder R. B. Curren B. F. Sikder M. A. A. Ullah A. Sebina I.. (2020). Long-lived regulatory T cells generated during severe bronchiolitis in infancy influence later progression to asthma. Mucosal Immunol. 13, 652–664. doi: 10.1038/s41385-020-0268-8, PMID: 32066837

[ref20] Ma F. Kouzoukas D. E. Meyer-Siegler K. L. Westlund K. N. Hunt D. E. Vera P. L. (2017). Disulfide high mobility group box-1 causes bladder pain through bladder toll-like receptor 4. BMC Physiol. 17:6. doi: 10.1186/s12899-017-0032-9, PMID: 28545586 PMC5445386

[ref21] Mazzone S. B. Reynolds S. M. Mori N. Kollarik M. Farmer D. G. Myers A. C.. (2009). Selective expression of a sodium pump isozyme by cough receptors and evidence for its essential role in regulating cough. J. Neurosci. 29, 13662–13671. doi: 10.1523/JNEUROSCI.4354-08.2009, PMID: 19864578 PMC3849768

[ref22] Mazzone S. B. Tian L. Moe A. A. K. Trewella M. W. Ritchie M. E. McGovern A. E. (2020). Transcriptional profiling of individual airway projecting vagal sensory neurons. Mol. Neurobiol. 57, 949–963. doi: 10.1007/s12035-019-01782-8, PMID: 31630330

[ref23] Mazzone S. B. Undem B. J. (2016). Vagal afferent innervation of the airways in health and disease. Physiol. Rev. 96, 975–1024. doi: 10.1152/physrev.00039.2015, PMID: 27279650 PMC4982036

[ref24] McGovern A. E. Davis-Poynter N. Yang S. K. Simmons D. G. Farrell M. J. Mazzone S. B. (2015). Evidence for multiple sensory circuits in the brain arising from the respiratory system: an anterograde viral tract tracing study in rodents. Brain Struct. Funct. 220, 3683–3699. doi: 10.1007/s00429-014-0883-9, PMID: 25158901

[ref25] McGovern A. E. Short K. R. Kywe Moe A. A. Mazzone S. B. (2018). Translational review: neuroimmune mechanisms in cough and emerging therapeutic targets. J. Allergy Clin. Immunol. 142, 1392–1402. doi: 10.1016/j.jaci.2018.09.004, PMID: 30409248

[ref26] Meijering E. Jacob M. Sarria J. C. Steiner P. Hirling H. Unser M. (2004). Design and validation of a tool for neurite tracing and analysis in fluorescence microscopy images. Cytometry A 58, 167–176. doi: 10.1002/cyto.a.20022, PMID: 15057970

[ref27] Merianda T. T. Coleman J. Kim H. H. Kumar Sahoo P. Gomes C. Brito-Vargas P.. (2015). Axonal amphoterin mRNA is regulated by translational control and enhances axon outgrowth. J. Neurosci. 35, 5693–5706. doi: 10.1523/JNEUROSCI.3397-14.2015, PMID: 25855182 PMC4388927

[ref28] O'Connell F. Springall D. R. Moradoghli-Haftvani A. Krausz T. Price D. Fuller R. W.. (1995). Abnormal intraepithelial airway nerves in persistent unexplained cough? Am. J. Respir. Crit. Care Med. 152, 2068–2075. doi: 10.1164/ajrccm.152.6.8520777, PMID: 8520777

[ref29] Ollerenshaw S. L. Jarvis D. Sullivan C. E. Woolcock A. J. (1991). Substance P immunoreactive nerves in airways from asthmatics and nonasthmatics. Eur. Respir. J. 4, 673–682. PMID: 1716217

[ref30] Park C. K. Xu Z. Z. Berta T. Han Q. Chen G. Liu X. J.. (2014). Extracellular microRNAs activate nociceptor neurons to elicit pain via TLR7 and TRPA1. Neuron 82, 47–54. doi: 10.1016/j.neuron.2014.02.011, PMID: 24698267 PMC3982230

[ref31] Parker T. M. Nguyen A. H. Rabang J. R. Patil A. A. Agrawal D. K. (2017). The danger zone: systematic review of the role of HMGB1 danger signalling in traumatic brain injury. Brain Inj. 31, 2–8. doi: 10.1080/02699052.2016.1217045, PMID: 27819487 PMC5610113

[ref32] Reznikov L. R. Meyerholz D. K. Adam R. J. Abou Alaiwa M. Jaffer O. Michalski A. S.. (2016). Acid-sensing ion channel 1a contributes to airway hyperreactivity in mice. PLoS One 11:e0166089. doi: 10.1371/journal.pone.0166089, PMID: 27820848 PMC5098826

[ref33] Rong L. L. Trojaborg W. Qu W. Kostov K. Yan S. D. Gooch C.. (2004). Antagonism of RAGE suppresses peripheral nerve regeneration. FASEB J. 18, 1812–1817. doi: 10.1096/fj.04-1899com, PMID: 15576484

[ref34] Saleh A. Smith D. R. Tessler L. Mateo A. R. Martens C. Schartner E.. (2013). Receptor for advanced glycation end-products (RAGE) activates divergent signaling pathways to augment neurite outgrowth of adult sensory neurons. Exp. Neurol. 249, 149–159. doi: 10.1016/j.expneurol.2013.08.018, PMID: 24029001

[ref35] Shapiro C. O. Proskocil B. J. Oppegard L. J. Blum E. D. Kappel N. L. Chang C. H.. (2021). Airway sensory nerve density is increased in chronic cough. Am. J. Respir. Crit. Care Med. 203, 348–355. doi: 10.1164/rccm.201912-2347OC, PMID: 32809840 PMC7874308

[ref36] Shibasaki M. Sasaki M. Miura M. Mizukoshi K. Ueno H. Hashimoto S.. (2010). Induction of high mobility group box-1 in dorsal root ganglion contributes to pain hypersensitivity after peripheral nerve injury. Pain 149, 514–521. doi: 10.1016/j.pain.2010.03.023, PMID: 20392563

[ref37] Simpson J. Loh Z. Ullah M. A. Lynch J. P. Werder R. B. Collinson N.. (2020). Respiratory syncytial virus infection promotes necroptosis and HMGB1 release by airway epithelial cells. Am. J. Respir. Crit. Care Med. 201, 1358–1371. doi: 10.1164/rccm.201906-1149OC, PMID: 32105156

[ref400] Szabo-Pardi T. A. Barron L. R. Lenert M. E. Burton M. D. (2021). Sensory Neuron TLR4 mediates the development of nerve-injury induced mechanical hypersensitivity in female mice. Brain. Behav. Immun. doi: 10.1016/j.bbi.2021.06.011, PMID: 34174335 PMC8453057

[ref38] Trankner D. Hahne N. Sugino K. Hoon M. A. Zuker C. (2014). Population of sensory neurons essential for asthmatic hyperreactivity of inflamed airways. Proc. Natl. Acad. Sci. U. S. A. 111, 11515–11520. doi: 10.1073/pnas.1411032111, PMID: 25049382 PMC4128113

[ref39] Ullah M. A. Loh Z. Gan W. J. Zhang V. Yang H. Li J. H.. (2014). Receptor for advanced glycation end products and its ligand high-mobility group box-1 mediate allergic airway sensitization and airway inflammation. J. Allergy Clin. Immunol. 134, 440–450. doi: 10.1016/j.jaci.2013.12.1035, PMID: 24506934

[ref40] Undem B. J. Taylor-Clark T. (2014). Mechanisms underlying the neuronal-based symptoms of allergy. J. Allergy Clin. Immunol. 133, 1521–1534. doi: 10.1016/j.jaci.2013.11.027, PMID: 24433703 PMC6483074

[ref41] Undem B. J. Zaccone E. McGarvey L. Mazzone S. B. (2015). Neural dysfunction following respiratory viral infection as a cause of chronic cough hypersensitivity. Pulm. Pharmacol. Ther. 33, 52–56. doi: 10.1016/j.pupt.2015.06.006, PMID: 26141017 PMC4532602

[ref42] Verzele N. A. J. Chua B. Y. Law C. W. Zhang A. Ritchie M. E. Wightman O.. (2021). The impact of influenza pulmonary infection and inflammation on vagal bronchopulmonary sensory neurons. FASEB J. 35:e21320. doi: 10.1096/fj.202001509R, PMID: 33660333

[ref43] West P. W. Canning B. J. Merlo-Pich E. Woodcock A. A. Smith J. A. (2015). Morphologic characterization of nerves in whole-mount airway biopsies. Am. J. Respir. Crit. Care Med. 192, 30–39. doi: 10.1164/rccm.201412-2293OC, PMID: 25906337 PMC4511424

[ref44] Yamasoba D. Tsubota M. Domoto R. Sekiguchi F. Nishikawa H. Liu K.. (2016). Peripheral HMGB1-induced hyperalgesia in mice: redox state-dependent distinct roles of RAGE and TLR4. J. Pharmacol. Sci. 130, 139–142. doi: 10.1016/j.jphs.2016.01.005, PMID: 26883456

[ref45] Yang H. Zeng Q. Silverman H. A. Gunasekaran M. George S. J. Devarajan A.. (2021). HMGB1 released from nociceptors mediates inflammation. Proc. Natl. Acad. Sci. U. S. A. 118:e2102034118. doi: 10.1073/pnas.2102034118, PMID: 34385304 PMC8379951

[ref46] Zaccone E. J. Lieu T. Muroi Y. Potenzieri C. Undem B. E. Gao P.. (2016). Parainfluenza 3-induced cough hypersensitivity in the Guinea pig airways. PLoS One 11:e0155526. doi: 10.1371/journal.pone.0155526, PMID: 27213574 PMC4877001

